# Transcriptome analyses provide insights into the phylogeny and adaptive evolution of the mangrove fern genus *Acrostichum*

**DOI:** 10.1038/srep35634

**Published:** 2016-10-26

**Authors:** Zhang Zhang, Ziwen He, Shaohua Xu, Xinnian Li, Wuxia Guo, Yuchen Yang, Cairong Zhong, Renchao Zhou, Suhua Shi

**Affiliations:** 1 State Key Laboratory of Biocontrol, Guangdong Provincial Key Laboratory of Plant Resources, Key Laboratory of Biodiversity Dynamics and Conservation of Guangdong Higher Education Institutes, Sun Yat-Sen University, Guangzhou, 510275, China; 2 Hainan Dongzhai Harbor National Nature Reserve, Haikou, 571129, China

## Abstract

The mangrove fern genus *Acrostichum* grows in the extremely unstable marine intertidal zone under harsh conditions, such as high salt concentrations, tidal rhythms and long-term climate changes. To explore the phylogenetic relationships and molecular mechanisms underlying adaptations in this genus, we sequenced the transcriptomes of two species of *Acrostichum*, *A. aureum* and *A. speciosum*, as well as a species in the sister genus, *Ceratopteris thalictroides*. We obtained 47,517, 36,420 and 60,823 unigenes for the three ferns, of which 24.39–45.63% were annotated using public databases. The estimated divergence time revealed that *Acrostichum* adapted to the coastal region during the late Cretaceous, whereas the two mangrove ferns from the Indo West-Pacific (IWP) area diverged more recently. Two methods (the modified branch-site model and the K_h_ method) were used to identify several positively selected genes, which may contribute to differential adaptation of the two *Acrostichum* species to different light and salt conditions. Our study provides abundant transcriptome data and new insights into the evolution and adaptations of mangrove ferns in the inhospitable intertidal zone.

The species of fern genus *Acrostichum* L. (Pteridaceae) are important components of mangrove community. They grow in the unstable marine intertidal zone, which is characterized by harsh conditions for plant growth, such as high salinity, tidal fluctuations and long-term climate changes^1^,^2^. Therefore, these species are referred as “mangrove ferns”^2^. This genus includes three species: *A. danaeifolium* Langsd. & Fisch., *A. aureum* L. and *A. speciosum* Willd^2^. *A. danaeifolium* and *A. speciosum* are restricted within the Atlantic East-Pacific (AEP) area and Indo West-Pacific (IWP) area, respectively, whereas *A. aureum* is the only species of mangroves that is widely distributed in both areas^3^. The three species of *Acrostichum* are all diploid, and *A. aureum* and *A. danaeifolium* have chromosome numbers of 2n = 60^4^,^5^ (Supplementary Information and Supplementary Fig. S1).

In the IWP area, *A. aureum* and *A. speciosum* often occur sympatrically but occupy different habitats with respect to light and salinity^6^. *A. aureum* is an upstream fern usually found in open, light-abundant habitats that are strongly influenced by fresh water, especially in mangrove forests that have been disturbed by human activities^7^, whereas *A. speciosum* is usually found in the shady mangrove understory, which is frequently flooded by tides^8^. *A. speciosum* appears to have greater salt tolerance than *A. aureum*^9^, which is corroborated by the differing Na^+^ and Cl^−^ levels in the roots and leaves of *A. aureum* and *A. speciosum*^10^. The differential adaptations of the two species to different light conditions are also reflected by their frond textures: *A. aureum* has thickly coriaceous fronds with a broadly rounded end, whereas *A. speciosum* has papery fronds with a pointed tip^6^. Although these plants prefer different environments, the two species can hybridize when their habitats overlap, especially in disturbed habitats^6^. However, only F1 hybrids have been found in the wild, suggesting strong postzygotic isolation between these species^6^. *Acrostichum* displays markedly differential adaptations to heterogeneous habitats, thus offering an excellent system in which to study adaptive evolution. For example, identifying positively selected genes in the genomes of *Acrostichum* species could contribute to our understanding of molecular mechanisms of adaptive evolution^11^,^12^. Additionally, *Acrostichum* is the only fern genus that grows in the intertidal zone, occupying a special position in ferns. By reconstructing the phylogeny of this genus and estimating divergence times, we can provide new insight on the origins of the genus.

To resolve these evolutionary questions, large amounts of molecular resources, such as whole-genome sequences and transcriptome data, are needed. Because fern species usually have large chromosome numbers and genome sizes, whole-genome sequencing is difficult; thus, complete genome sequences are not available for ferns, including *Acrostichum*^13^. RNA-seq is a relatively convenient choice because a large number of sequences can be obtained at low cost^13^. A number of ferns have been studied using this strategy, such as the bracken fern *Pteridium aquilinum*^14^, the fresh-water fern *Ceratopteris richardii*^15^, the Japanese climbing fern *Lygodium japonicum*^16^, as well as the fern species in the 1,000 Plants (1 KP) project^17^.

In this study, we sequenced, *de novo* assembled and annotated the transcriptomes of two mangrove fern species of *Acrostichum* (*A. aureum* and *A. speciosum*) and one species of its non-mangrove sister genus, *Ceratopteris thalictroides*. By combining the published chloroplast sequences and genomic data, we sought to 1) resolve the phylogenetic relationships in *Acrostichum*; 2) estimate the divergence times between *Acrostichum* and its sister genus *Ceratopteris*, as well as within the *Acrostichum* genus; and 3) detect candidate genes that are under positive selection in mangrove ferns.

## Results

### Transcriptome assembly and annotations

We obtained 22–26 million raw reads for the three fern species, from which 17–21 million clean reads were retrieved after quality control (Table 1). These reads were *de novo* assembled into 53,831, 41,661 and 69,929 contigs for *A. aureum*, *A. speciosum* and *C. thalictroides*, respectively, using Trinity^18^, a *de novo* transcriptome assembler (Table 1). After the redundancies were removed, 47,517, 36,420 and 60,823 contigs with N50 values of 1,136 bp, 1,687 bp and 787 bp, respectively, were treated as unigenes in the downstream analyses (Table 1). These unigenes were deposited in the NCBI GenBank under accession numbers GEEI00000000 (*A. aureum*), GEEJ00000000 (*A. speciosum*) and GEEK00000000 (*C. thalictroides*). The length distribution showed that 36.8–56.1% of the unigenes were longer than 500 bp (Supplementary Fig. S2). The GC content of *A. aureum* was 46.33%, which was slightly higher than that of *A. speciosum* (45.80%) and *C. thalictroides* (44.08%).

**Figure 1:**
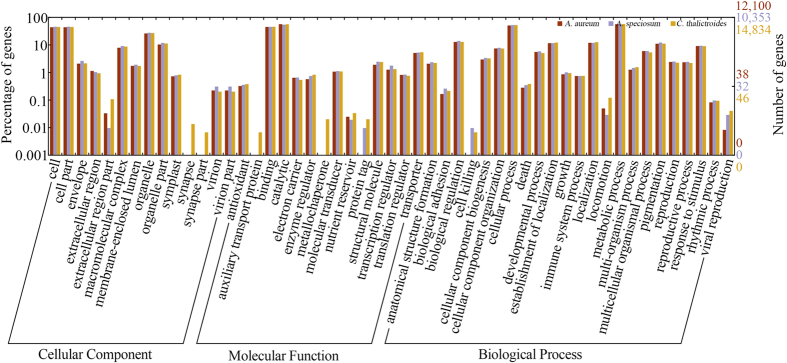
GO term (level 2) distribution for the transcriptomes of *A.* *aureum*, *A. speciosum* and *C. thalictroides*. In total, 12,100, 10,353 and 14,834 unigenes for *A. aureum*, *A. speciosum* and *C. thalictroides,* respectively, were assigned to at least one GO term and grouped into three main GO categories and 51 GO terms.

The functional annotations were performed based on similarity to the SwissProt protein database and NCBI non-redundancy protein database. A total of 35.89–58.35% unigenes returned a BLASTX hit above the e-value cut-off of 10^−6^ from these two databases (Table 2). Two annotation programs, Blast2GO^19^ and GOanna of Agbase^20^, were used for the functional annotations and Gene Ontology (GO) term retrievals. For *A. aureum*, *A. speciosum* and *C. thalictroides*, 12,100 (25.46%), 10,353 (28.43%) and 14,834 (24.39%) unigenes were annotated, respectively. The GO terms were assigned using Blast2GO (Table 2), and the distribution of level-2 GO terms was plotted (Fig. 1). A total of 4,501, 3,642 and 6,143 unigenes were assigned to 124, 122 and 127 KEGG pathways, respectively, for the three species (Table 2). A total of 42.05% of *A. aureum*, 45.63% of *A. speciosum* and 38.95% of *C. thalictroides* unigenes were matched to the annotation results from Agbase, a genomic database that contains functional annotations of agricultural species (Table 2). Detailed information on the functional annotations as well as the identified transcription factors and simple sequence repeats (SSRs) is provided in the Supplementary Information (Supplementary text, Supplementary Figs S3–S5 and Supplementary Tables S1–S10).

### Phylogenetic analysis based on chloroplast genes and transcriptome data

**Figure 2:**
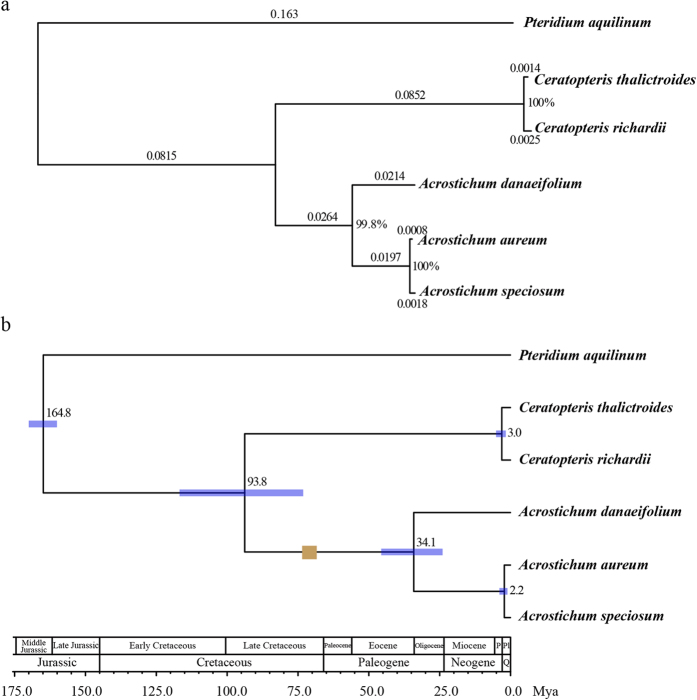
Phylogenetic tree and divergence time estimation based on the sequences of four chloroplast genes. (**a**) Phylogenetic tree. Branch lengths are marked on each branch. Numbers near each node are the bootstrap values. (**b**) Divergence time is based on the sequences of four chloroplast genes. The brown box indicates the earliest fossil record of *Acrostichum* (Maastrichtian in the late Cretaceous^23^, approximately 66.0–72.1 Mya). The blue bars represent the 95% confidential intervals. Q, Quaternary; P, Pliocene; Pl, Pleistocene.

We concatenated the four chloroplast genes (*atpA*, *atpB*, *rbcl* and *rps4*) from six species (three species of *Acrostichum*, two species of *Ceratopteris* and the out-group species *Pteridium aquilinum*) to reconstruct the phylogenetic tree, and our results showed that each node of the tree was highly supported (Fig. 2a). The three species of *Acrostichum* formed a monophyletic clade, with *A. danaeifolium* diverging first and *A. aureum* and *A. speciosum* representing sister species. The divergence time between *Acrostichum* and *Ceratopteris* was estimated at approximately 93.8 Mya (Fig. 2b). Within *Acrostichum*, *A. danaeifolium* diverged from the other two species approximately 34.1 Mya, whereas the divergence between *A. aureum* and *A. speciosum* was much more recent (2.2 Mya).

**Figure 3:**
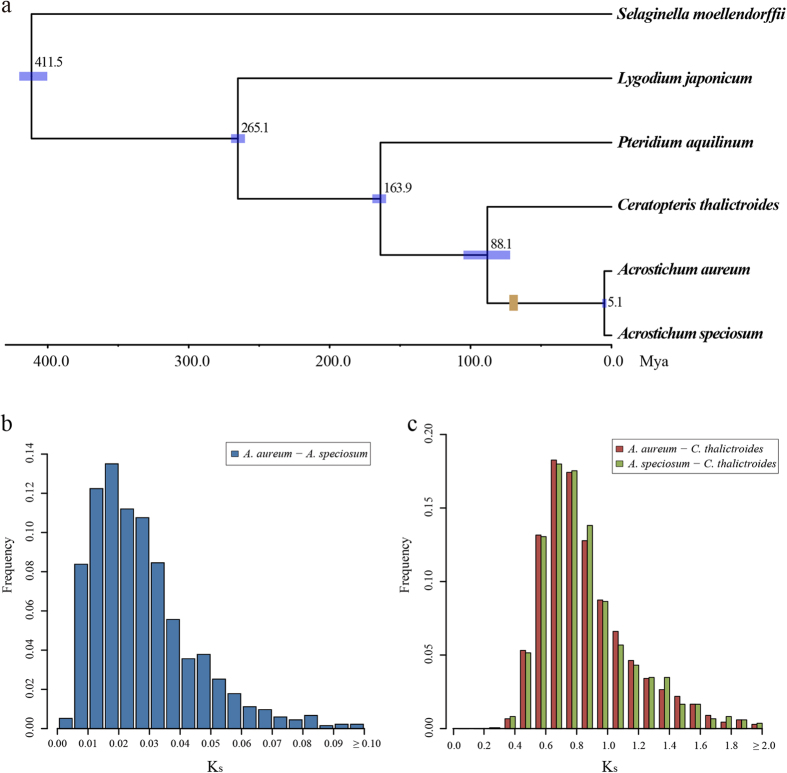
Divergence time estimated based on transcriptome data. (**a**) Divergence estimation based on single-copy orthologs. The brown box indicates the earliest fossil record of *Acrostichum.* (**b**) K_s_ distribution between *A. aureum* and *A. speciosum*. (**c**) K_s_ distribution between *Acrostichum* and *Ceratopteris*.

A total of 18,500 gene families were generated from the genomic and transcriptomic data of six species (*A. aureum*, *A. speciosum*, *C. thalictroides*, *P. aquilinum*, *Lygodium japonicum* and *Selaginella moellendorffii*) using OrthoMCL software^21^. From these data, 1,364 single-copy orthologs were chosen for phylogeny reconstruction and divergence-time estimation (Supplementary Table S11). Phylogenetic analyses were consistent with earlier studies of the phylogeny of Pteridaceae^22^, which suggested that *Ceratopteris* and *Acrostichum* last shared a common ancestor 88.1 million years ago in the late Cretaceous. The sister species *A. aureum* and *A. speciosum* diverged approximately 5.1 Mya. In addition, we calculated the K_s_ (synonymous substitution rate) of each ortholog identified before. These values reflected the relationships of *Acrostichum* and *Ceratopteris*. The peak of the distribution of the pairwise K_s_ values between *A. aureum* and *A. speciosum* was approximately 0.02 (Fig. 3b), suggesting a minor divergence between these two species; whereas, the peaks of the distribution of pairwise K_s_ values between each of the two *Acrostichum* species and *C. thalictroides* were approximately 0.7 (Fig. 3c), indicating a large divergence between the sister genera *Acrostichum* and *Ceratopteris*.

Combined with the earliest fossil record of *Acrostichum* (Maastrichtian in the late Cretaceous^23^, 66.0–72.1 Mya), these results suggest that this genus might have diverged from its sister genus during the late Cretaceous. The divergence of the AEP fern *A. danaeifolium* at approximately 34.1 Mya might have triggered by the Eocene/Oligocene climatic crisis^24^.

### Putative positively selected genes (PSGs) detected with the branch-site model and the K_h_ method

Genes under positive selection are often identified using the ratio of the nonsynonymous substitution rate to the synonymous substitution rate (K_a_/K_s_)^25^. A K_a_/K_s_ value that is significantly larger or smaller than 1 is interpreted as evidence of positive/purifying selection, and a K_a_/K_s_ equivalent to 1 indicates neutral evolution^25^. However, this method is stringent, and positive selection often acts on a few sites of a gene over a short interval^26^ and can be counteracted by negative selection at the remaining sites^27^. Therefore, two additional methods, the modified branch-site model^26^ and the K_h_ test^27^, were used in this study to detect candidate PSGs.

A total of 3,164 orthologs generated from the transcriptomes of four species (*A. aureum*, *A. speciosum*, *C. thalictroides* and *C. richardii*) were used to identify candidate PSGs using the modified branch-site model. We detected 27 and 31 PSGs in the branches of *A. aureum* and *A. speciosum* with p-values < 0.05 based on a Chi-square test. After the Benjamini-Hochberg correction^28^ was applied, only six and three genes for *A. aureum* and *A. speciosum*, respectively, remained. Because the Benjamini-Hochberg correction is a stringent correction used to reduce the false-positive rate, and may remove true PSGs, we retained all candidate PSGs from before the Benjamini-Hochberg correction for the functional annotation. These PSGs were involved in metabolic processes, RNA or DNA binding and specific enzymatic reactions that play a role in responses to light and salt stresses (see Discussion and Supplementary Tables S12–S13 for detailed information).

**Figure 4:**
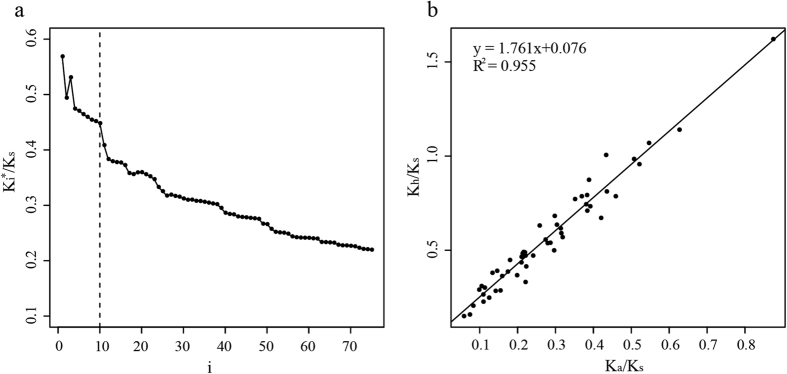
Results of K_i_^*^/K_s_. (**a**) K_i_^*^/K_s_ versus i. i denotes the universal ranking of each amino acid change and range from the most exchangeable pairs (i = 1) to the least exchangeable pairs (i = 75)^67^. K_75_^*^/K_s_ is equivalent to K_a_/K_s_. K_10_^*^/K_s_ is almost twice the value of K_a_/K_s_. (**b**) Scatter plot of K_h_/K_s_ versus K_a_/K_s_ for 53 supergenes between *A. aureum* and *A. speciosum*. Each supergene contains 100 orthologs with similar K_a_ values.

Using the K_h_ method, 7,379 orthologs from *A. aureum* and *A. speciosum* were obtained. We observed 16,183 amino acid changes between these two species, and 15,181 of which were elementary amino acid changes (changed by 1 bp). The top 10–12 classes had 3,961–5,232 elementary amino acid changes that accounted for 25–32% of the total changes. The ratio of K_i_^*^/K_s_ (K_i_^*^ is the cumulative rate of the first i classes of amino acid substitution.) versus the i-th class of amino acid changes is plotted in Fig. 4a. The value of K_10_^*^/K_s_ was almost twice that of K_a_/K_s_, which is consistent with the “twofold approximation” pattern that has been observed in yeast, primates and rodents^27^. This twofold pattern was also supported by 53 supergenes that combined 100 orthologs with similar K_a_ values and presented a regression line slop of 1.76 (Fig. 4b). We defined K_10_^*^ as K_h_, which refers to a class of highly exchangeable substitutions, after the method of Tang and Wu^27^. The standard K_h_/K_s_ > 1 may be a reasonable standard similar to K_a_/K_s_ > 1 for use in identifying genes under positive selection^27^. In total, 227 genes presented K_a_/K_s_ > 1, but only one was significant, whereas the K_h_/K_s_ values of 15 genes were significantly greater than 1 (Table 3, Supplementary Table S14 and Supplementary Fig. S6). Three genes were involved in responses to light, and the others had functions in binding, kinase activity, metabolism, etc.(Table 3 and Supplementary Table S14).

## Discussion

Previous studies of *Acrostichum* have focused on its physiology, morphology and ecology^7^,^8^, and only one recent study has reported natural hybridization between two *Acrostichum* species in the IWP region^6^. To date, there have been no published genomic data for *Acrostichum*, and the EST sequences in the NCBI database are too limited to address evolutionary questions, such as the origin of the genus and the identification of genes under positive selection. The transcriptome data for *Acrostichum* that were developed in this study provide new resources for mangrove ferns.

In this study, we used chloroplast genes and thousands of orthologous genes from transcriptomic/genomic data to estimate the divergence time between *Acrostichum* and its sister genus, and between species within *Acrostichum*. Based on the results from the two datasets, the *Acrostichum* genus diverged from the closely related *Ceratopteris* approximately 88.1 Mya and the AEP fern *A. danaeifolium* diverged from the other IWP ferns approximately 34.1 Mya. The earliest known *Acrostichum* fossil is a permineralized aerial stem with petioles and roots that was identified in the Deccan Intertrappean beds of India^23^. This fossil is dated to the Maastrichtian in the late Cretaceous, which is approximately 66.0–72.1 million years before present. Aerenchyma tissue is a morphological feature considered to be an adaptation to aquatic life in both *Ceratopteris* and *Acrostichum*^29^, and has been found in the roots of the *Acrostichum* fossil, which suggests a coastal palaeoenvironment^23^. Fossils of coastal palms, mangroves and marsh plants have also been found in this region or nearby^23^, indicating that the ancestor of *Acrostichum* had grown in and adapted to the coastal region by at least the late Cretaceous. Mangroves were pantropic by the Eocene and appeared to have originated during the Paleocene^24^. Our results revealed that *Acrostichum* is one of the oldest members of the mangrove ecosystem and dates to the late Cretaceous along with the mangrove palm *Nypa*^30^,^31^. It is reported that there are 10,560 extant fern species which belong to 215 genera^32^, and this large species richness may result from a burst of fern diversification in the Cenozoic (from 66 million years ago to present day)^33^. However, *Acrostichum* had only three extant species although it diverged from its sister genus since 88.1 Mya, which may imply that the intertidal zone might be an extremely inhospitable environment for plants to survive. *A. danaeifolium*, the species restricted to the AEP area, diverged approximately 34.1 Mya (in the late Oligocene) and was traced back to the Eocene/Oligocene climate crisis^24^.

For the divergence time between *A. aureum* and *A. speciosum*, the estimate based on the chloroplast genes (2.2 Mya) is more recent than the one based on transcriptome data (5.1 Mya). This discrepancy in estimates of divergence time may be caused by ancient chloroplast capture through interspecific introgression and hybridization. In the early stage of their divergence, the interspecific hybridization and subsequent backcrossing for several generations would create a new combination of mainly one species’ nuclear genome and completely another species’ chloroplast genome, due to maternal inheritance and absence of recombination in chloroplast DNA^34^. This ancient chloroplast capture would influence and reduce the divergence of chloroplast genome; thus, divergence time estimates based on chloroplast genes would be more recent. In addition, only four genes were used to estimate the divergence time; therefore, variations between the sister species may not sufficiently reflect their divergence. Additional chloroplast genes should be used in future studies to increase the accuracy of divergence estimate.

*A. aureum* and *A. speciosum* diverged from each other very recently in the IWP area and prefer different habitats with respect to salt and light conditions. To reveal the molecular mechanisms underlying these adaptations, we used two methods to detect candidate PSGs. The modified branch-site model identifies PSGs based on a likelihood ratio test of models for the foreground lineage under selection and without selection. This approach has been widely used for genomic data and exploits the advances in genome sequencing technology that have been made in recent years^35^,^36^. Many previous works showed that different amino acid pairs have different exchangeability^37^,^38^; therefore, amino acid pairs with high exchangeability could be more sensitive indicators of positive selection signals that are hidden by purifying selection. Tang and Wu developed a new method using the K_h_ statistic, which is the cumulative rate of nonsynonymous substitutions for the 10 most exchangeable classes, instead of the K_a_ statistic^27^. The value of K_h_ is approximately twice that of K_a_ in mangrove ferns, and a similar pattern is observed in yeast and animals^27^. A ratio of K_h_/K_s_ significantly greater than 1 is a potential new criterion for detecting positive selection^27^.

Certain genes detected by the modified branch-site model were related to salt and light stress responses. For example, the SKIP (SNW/SKI-interacting protein) gene was identified under positive selection when *A. aureum* and *A. speciosum* were set as the foreground branch. This gene could improve the abiotic stress resistance via the regulation of abscisic acid signal transduction^39^, contribute to cytokinin-regulated leaf initiation^40^, and participate in photomorphogenesis by regulating the signaling of cell cycle^41^. The 27 PSGs in the lineage of *A. aureum* include AtBAG4 (Bcl-2-associated athanogene 4), an anti-apoptotic gene that significantly enhances the salt tolerance of rice^42^; adenylate kinase (ADK), a kinases of the SnRK1-ADK complexes that participates managing biotic and abiotic stresses and maintaining energy homeostasis^43^; and phosphomannomutase (PMM), which is required for the GDP-mannose biosynthesis, ascorbic acid biosynthesis and N-glycosylation and plays an important role in temperature adaptability^44^,^45^. Of the 31 PSGs in the *A. speciosum* lineage, two are involved in the response to light. Photosystem II core phosphatase (PBCP) is important for effective dephosphorylation of the core subunits of photosystem II and may influence the state transitions between photosystem I and photosystem II^46^. Ribosomal protein L10B (RPL10B) may participate in the responses to different stresses, especially to ultraviolet B^47^ (see Supplementary Tables S12–S13 for detailed information on each PSG).

Among the 15 positively selected genes identified using the K_h_ method, one gene was annotated as a phototropic-responsive NPH3 family protein, which function in signal transduction of phototropic response^48^,^49^. *A. aureum* is often found in open places with full light, while *A. speciosum* usually grows under mangrove forests. As the two *Acrostichum* species have different preferences for light, this gene may contribute to their differential adaptations to different light conditions. In addition, two genes encoded as DHNA-CoA (1,4-dihydroxy-2-naphthoyl-CoA) thioesterase may contribute to the biosynthesis of phylloquinone (vitamin K_1_)^50^, an electron acceptor of the electron transport chain in Photosystem I.

PSGs did not overlap between the two methods, which may be related to the different assumptions of the methods. The modified branch-site model uses the likelihood ratio test to detect the PSGs on a given branch, whereas the K_h_ method examines highly exchanged amino acid pairs for evidence of positive selection in order to reduce the influence of purifying selection. The K_h_ method can identify genes under selection but cannot determine the direction of selection. Although each method found a subset of PSGs, all of the candidate PSGs identified by the two methods were related to responses to light, including an elector acceptor and proteins involved in photosystem state transitions, phototropic responses and UV stress responses. Based on the recent divergence between *A. aureum* and *A. speciosum* and their different preferences for light, these PSGs might be important for the adaptation and speciation of the two mangrove ferns.

In summary, we sequenced the transcriptomes for two species of the mangrove fern genus *Acrostichum* and one species of its sister genus *Ceratopteris*, providing new genomic resources for both ferns and mangroves. Phylogenetic reconstruction and divergence time estimation based on both transcriptome data and chloroplast genes revealed that *Acrostichum* adapted to the coastal region during the late Cretaceous, whereas the two mangrove ferns in the Indo West-Pacific (IWP) region diverged recently. Positively selected genes, such as SKIP gene, NPH3 family protein, etc., were detected by the modified branch-site model and the K_h_ method, which may contribute to differential adaptations of *Acrostichum* species to different intertidal habitats.

## Methods

### Sampling, RNA extraction and sequencing

Samples of *A. aureum* and *A. speciosum* were collected from Nansha, Guangzhou, Guangdong (22°48′34.57″N, 113°34′56.38″E) and Qinglan Harbour, Wenchang, Hainan (19°37′33.11″N, 110°47′33.94″E), respectively. *C. thalictroides* was cultivated in the greenhouse of Sun Yat-sen University (Supplementary Table S15). Young leaves of each species were harvested to extract total RNA by the modified CTAB method^51^. cDNA library construction and sequencing were conducted by the Beijing Genome Institute (BGI, Shenzhen, China). Paired-end reads were obtained using the Illumina HiSeq2000 sequencing platform (Illumina, San Diego, USA). After we filtered the sequence adaptors, we deposited all raw reads into the NCBI short read archive (SRA) repository under accession numbers SRR1822234 (*A. aureum*), SRR1822235 (*A. speciosum*) and SRR1822236 (*C. thalictroides*).

### Data filtering, *de novo* assembly and functional annotation

The raw reads were first trimmed using the DynamicTrim program of the SolexaQA package^52^ at a quality threshold of 20. We then filtered reads less than 50 bp long using the LengthSort program of the same package. The clean reads of *A. aureum*, *A. speciosum* and *C. thalictroides* were *de novo* assembled into contigs using the short read assembly program Trinity^18^ under the default settings except ‘min_kmer-cov = 2’. Then, the programs TGICL^53^ and CDHIT^54^ were used to remove redundant contigs under the default parameters. We mapped the clean reads to these contigs and calculated the mean coverage for each contig. Contigs with an average depth of less than two were discarded, and the remaining contigs were treated as unigenes in the subsequent analyses.

To determine the functional categories of the transcripts of three fern species, a BLASTX search was performed against the NCBI non-redundant (NR) protein database and SwissProt database^55^ (http://web.expasy.org/docs/swiss-prot_guideline.html) with an e-value cut-off of 10^−6^. The results of the NR BLASTX hits were processed with Blast2GO software^19^ (v.3.0.9 PRO) to assign functional annotations and retrieve the GO terms. The distribution of the level-2 GO terms for the three categories, biological processes, molecular functions and cellular components, was plotted in WEGO^56^. We conducted a pathway analysis against the Kyoto Encyclopedia of Genes and Genomes (KEGG) database using Blast2GO. The three transcriptomes were annotated with the SwissProt database using GOanna of Agbase^20^ (http://www.agbase.msstate.edu/cgi-bin/tools/GOanna.cgi) with a cut-off e-value of 10^−6^.

### Phylogenetic analyses and divergence time estimation

To infer the phylogenetic relationships and divergence times within the *Acrostichum* genus, four chloroplast genes (*atpA, atpB, rbcl* and *rps4*) from six species (three species of *Acrostichum*, two species of *Ceratopteris* and the out-group species *Pteridium aquilinum*) were used. The chloroplast sequences of *Acrostichum danaeifolium*, *Ceratopteris richardii* and *Pteridium aquilinum* were downloaded from the NCBI GenBank (Supplementary Table S16), and the corresponding genes of *A. aureum*, *A. speciosum* and *C. thalictroides* were obtained from transcriptome data using BLASTN with a cut-off e-value of 10^−6^. Chloroplast genes were aligned by MUSCLE^57^ and concatenated into one supergene. Before the phylogeny was reconstructed, an appropriate nucleotide-substitution model was selected from 88 substitution models using the jModelTest2 program^58^. The phylogenetic tree was built using PhyML^59^ with the best-fit model (GTR + G) and 1,000 replicates of the bootstrap analysis. The divergence time of each node was calculated by MCMCTree with the PAML 4.8 package^60^ using ‘seq like (usedata = 1)’, ‘HKY85 + gamma (model = 4; alpha = 0.5)’ and ‘independent rates (clock = 2)’. The time constraint between *Pteridium* and the ancestor of *Acrostichum* and *Ceratopteris* was set at 160–170 Mya according to the results of Schuettpelz *et al*.^33^.

A phylogenetic analysis was also performed on the orthologous genes that were generated from the genome or transcriptome data of six species, including *A. aureum*, *A. speciosum*, *C. thalictroides*, *P. aquilinum*, *Lygodium japonicum* and *Selaginella moellendorffii*. The transcriptome data of *P. aquilinum*, *L. japonicum* and *S. moellendorffii* were downloaded from Der *et al*.^14^, the *Lygodium japonicum* Transcriptome Database (http://bioinf.mind.meiji.ac.jp/kanikusa/) and Phytozome^61^,^62^, respectively. For each species pair, an all-versus-all sequence similarity search was conducted on the protein sequence using BLASTP with an e-value cut-off of 10^−10^ and an identity threshold of 40%. The BLASTP results were imported into OrthoMCL software^21^ for orthologous group clustering under the default settings. The protein sequences of the single-copy orthologs were aligned with MUSCLE^57^ and then converted to nucleotides with Pal2nal^63^. Alignments longer than 150 bp were retained for the phylogeny reconstruction and dating. JModeltest2, PhyML and MCMCTree were applied for model selection, phylogenetic tree reconstruction and divergence time calculation as described above. We employed two additional time constraints when dating the divergence time between *S. moellendorffii* and the true ferns (400–420 Mya)^64^,^65^ and between Schizaeoid ferns and the core leptosporangiates (260–270 Mya)^33^. The K_s_ values for the orthologs were also estimated using the KaKs-Calculator^66^ with the YN model to examine the distance between these species. The alignments used in the phylogenetic analyses were deposited in TreeBASE (http://purl.org/phylo/treebase/phylows/study/TB2:S19541).

### Identification of candidate positively selected genes (PSGs)

We applied two methods to identify putative PSGs in *Acrostichum*: the improved branch-site model^26^ implemented in codeml of the PAML 4.8 package^60^ and the K_h_ method developed by Tang and Wu^27^.

The modified branch-site model was used to identify PSGs along the branches of *A. aureum* and *A. speciosum* based on the 3,164 single-copy orthologs of four species (*A. aureum*, *A. speciosum*, *C. thalictroides* and *C. richardii*). The transcriptome data of *C. richardii* were downloaded from Bushart *et al*.^15^. *A. aureum* and *A. speciosum* were set as the foreground branch separately, and then a likelihood ratio test was performed to compare the null model (no signal of positive selection) to the alternative model (positive selection on certain codons)^26^. The ancestral branch of *A. aureum* and *A. speciosum* was not included in this study. The Benjamini-Hochberg correction^28^ with a false-discovery rate of 5% was used for multiple testing. We also annotated these genes based on the homologues of *Arabidopsis* in The Arabidopsis Information Resource (TAIR, https://www.arabidopsis.org/).

The orthologs between *A. aureum* and *A. speciosum* were assessed using OrthoMCL software^21^ and aligned as described above. The universal evolutionary index (EI(i), i = 1–75) of Tang *et al*.^67^ ranked the 75 elementary amino acid changes where the codon differed by 1 bp from the most exchangeable (i = 1) to the least exchangeable class (i = 75). To determine whether *Acrostichum* has the “twofold approximation” pattern reported in Tang and Wu^27^, we first concatenated all genes into one supergene to calculate the overall values of the cumulative rate of the first i types of amino acid changes (K_i_^*^), synonymous substitution rates (K_s_) and nonsynonymous substitution rates (K_a_). We counted the observed synonymous substitutions (N_s_) and total synonymous sites (L_s_) of pairwise alignments to calculate the K_s_ value. Then, the observed substitutions and total sites of each class of elementary amino acid change (N_i_ and L_i_, respectively, i = 1, 2, …, 75) were counted to calculate the K_i_ value. The ratio of the transition rate to the transversion rate (kappa) was estimated from fourfold degenerate sites using baseml in the Paml 4.8 package. The values of K_s_, K_i_ (the nonsynonymous substitution rate of the ith-type amino acid change) and K_i_^*^ were calculated using the method of Jukes and Cantor^68^ for multiple hit corrections. The K_75_^*^ value was equivalent to K_a_ in this calculation. To detect this twofold pattern in genes with different K_a_ values, 5,304 genes with K_a_ > 0 were sorted in descending order, and then every 100 genes were concatenated to conduct the same estimations. To identify positively selected gene, the K_a_, K_s_ and K_i_^*^ values of each ortholog were estimated using the same method. According to Tang and Wu^27^, K_10_^*^ was defined as K_h_, the class of high-exchangeable substitutions, and a threshold of K_h_/K_s_ significantly greater than 1 was used as the criterion for isolating PSGs. Fisher’s exact test implemented in R was used to test for significance. We removed genes with K_a_ > 0.05, K_s_ > 0.08 or K_s_ < 0.005 and then treated the remaining genes with K_h_/K_s_ > 1 and p-value < 0.05 as candidate PSGs.

## Additional Information

**Accession codes:** All raw reads were deposited in the NCBI short read archive (SRA) repository under accession numbers SRR1822234 (*A. aureum*), SRR1822235 (*A. speciosum*) and SRR1822236 (*C. thalictroides*). The unigenes of three species were deposited in NCBI GenBank under the accession numbers of GEEI00000000 (*A. aureum*), GEEJ00000000 (*A. speciosum*) and GEEK00000000 (*C. thalictroides*).

**How to cite this article**: Zhang, Z. *et al*. Transcriptome analyses provide insights into the phylogeny and adaptive evolution of the mangrove fern genus *Acrostichum*. *Sci. Rep.* **6**, 35634; doi: 10.1038/srep35634 (2016).

**Publisher’s note:** Springer Nature remains neutral with regard to jurisdictional claims in published maps and institutional affiliations.

## Supplementary Material

Supplementary Information

Supplementary Table S1

Supplementary Table S2

Supplementary Table S3

Supplementary Table S4-S16

## Figures and Tables

**Table 1:** A summary of the sequencing and assembly of *A. aureum*, *A. speciosum* and *C. thalictroides*.

	*A. aureum*	*A. speciosum*	*C. thalictroides*
Total number of raw reads	22,296,934 × 2	26,070,890 × 2	22,344,227 × 2
Total number of clean reads	17,139,763 × 2	21,021,468 × 2	17,764,317 × 2
Reads length (bp)	90	100	90
Total number of contigs	53,831	41,661	69,929
Total number of unigenes	47,517	36,420	60,823
Mean length (bp)	731	1,000	576
Median length (bp)	448	625	376
N50 value (bp)	1,136	1,687	787
Longest unigene (bp)	10,894	8,743	9,609
GC content	46.33%	45.80%	44.08%

The N50 value refers to the length at which the sum of all contigs of that length or longer accounts for 50% of the total length of the assembly.

**Table 2:** Functional annotations of the *de novo* transcriptomes for *A. aureum*, *A. speciosum* and *C. thalictroides*.

	*A. aureum*	*A. speciosum*	*C. thalictroides*
SwissProt-blast	19,304 (40.63%)	17,002 (46.68%)	21,832 (35.89%)
NR-blast	25,484 (53.63%)	21,250 (58.35%)	29,413 (48.36%)
NR-annotation	12,100 (25.46%)	10,353 (28.43%)	14,834 (24.39%)
KEGG	4,501 (9.47%)	3,642 (10.00%)	6,143 (10.10%)
Agbase-GOanna	19,980 (42.05%)	16,619 (45.63%)	23,690 (38.95%)

NR: NCBI non-redundant protein database. KEGG: Kyoto Encyclopedia of Genes and Genomes. Agbase-GOanna: http://www.agbase.msstate.edu/cgi-bin/tools/GOanna.cgi.

**Table 3:** A Summary of positively selected genes (PSGs) identified by the K_h_ method.

Sequence ID of *A. aureum*	Sequence ID of *A. speciosum*	K_h_/K_s_ (p-value)	K_a_/K_s_ (p-value)	Accession numbers of homologs in *Arabidopsis*	Function description in *Arabidopsis*
Aau_c10874_g1_i1	Asp_c9691_g1_i1	5.350 (0.007)	1.587 (0.297)	—	—
Aau_c13092_g1_i1	Asp_c4172_g1_i1	5.563 (0.006)	0.834 (0.738)	AT5G48800.1	Phototropic-responsive NPH3 family protein
Aau_c7880_g1_i1	Asp_c14498_g1_i1	6.131 (0.008)	1.461 (0.402)	AT2G28470.1	Beta-galactosidase 8
Aau_CL1753Contig1	Asp_c8981_g1_i1	4.196 (0.025)	1.246 (0.478)	AT4G29050.1	Concanavalin A-like lectin protein kinase family protein
Aau_CL1913Contig1	Asp_c11956_g2_i1	9.072 (0.007)	2.349 (0.205)	—	—
Aau_c10529_g1_i1	Asp_c15777_g2_i1	5.702 (0.005)	1.866 (0.184)	—	—
Aau_c30123_g1_i3	Asp_c16680_g2_i1	10.663 (0.023)	3.708 (0.165)	AT3G06920.1	Tetratricopeptide repeat (TPR)-like superfamily protein
Aau_c32708_g2_i1	Asp_c10898_g1_i3	10.018 (0.043)	1.304 (0.648)	AT1G48320.1	DHNA-CoA Thioesterase 1, DHNAT1
Aau_c17860_g2_i1	Asp_c29065_g1_i1	7.624 (0.026)	1.000 (0.687)	AT2G20700.1	LORELEI-LIKE-GPI ANCHORED PROTEIN 2
Aau_c13859_g1_i1	Asp_c15219_g1_i1	5.156 (0.025)	1.629 (0.333)	AT3G03080.1	Zinc-binding dehydrogenase family protein
Aau_c11978_g1_i2	Asp_c2275_g1_i1	4.229 (0.039)	0.934 (0.678)	—	—
Aau_c30536_g2_i1	Asp_c10452_g1_i1	2.742 (0.049)	0.799 (0.802)	AT3G17900.1	unknown protein
Aau_c30812_g1_i1	Asp_c21021_g1_i1	10.102 (0.041)	1.622 (0.553)	AT5G13510.1	Ribosomal protein L10 family protein
Aau_c32708_g2_i2	Asp_c10898_g1_i2	10.946 (0.002)	1.513 (0.459)	AT1G48320.1	DHNA-CoA Thioesterase 1, DHNAT1
Aau_c33212_g2_i1	Asp_c16931_g3_i2	3.389 (0.001)	1.991 (0.006)	AT4G32300.1	S-domain-2 5
