# Supplementary material for: Transcriptome analyses provide insights into the phylogeny and adaptive evolution of the mangrove fern genus Acrostichum
Source: Sci Rep. 2016 Oct 26;6:35634. doi: 10.1038/srep35634 (PMC5080628; doi:10.1038/srep35634)
Supplement: Supplementary Information [file srep35634-s1.pdf]

Supplementary Information of

Transcriptome analyses provide insights into the phylogeny and adaptive evolution of the mangrove fern genus *Acrostichum*

Zhang Zhang[1], Ziwen He[1], Shaohua Xu[1], Xinnian Li[1], Wuxia Guo[1], Yuchen Yang[1], Cairong Zhong[2], Renchao Zhou[1*] and Suhua Shi[1*]

[1] State Key Laboratory of Biocontrol, Guangdong Provincial Key Laboratory of Plant Resources, Key Laboratory of Biodiversity Dynamics and Conservation of Guangdong Higher Education Institutes, Sun Yat-Sen University, Guangzhou, 510275, China

[2] Hainan Dongzhai Harbor National Nature Reserve, Haikou, 571129, China

*Corresponding authors: lssssh@mail.sysu.edu.cn (S.S.) and zhrench@mail.sysu.edu.cn (R.Z.)

Supplementary Text

DNA content estimation

Previous studies showed that both *A. aureum* and *A. danaeifolium* were diploid with 2n = 60 (Lovis 1997; Macron *et al*., 2003). In contrast, *A. speciosum* was suggested as a polyploid because its n was approximately 58 (Tindale and Roy 2002). However, we highly suspect this result is wrong because of two lines of evidences. Zhang *et al.* (2013) showed that the spores of *A. aureum* and *A. speciosum* were similar in size. If *A. speciosum* is a polyploid, its spores should be larger than those of *A. aureum*. In addition, population genetics analyses (Zhang *et al.*, 2013) on three genes of *A. speciosum* revealed that no individual has more than two alleles at these loci, which also proved that *A. speciosum* should be diploid. To confirm the ploidy of *A. speciosum*, we estimated the DNA content of the transcriptome-sequenced individuals of *A. aureum* and *A. speciosum* using flow cytometry method according to the simplified two-step protocol of Dolezel *et al.* (2007). Samples were analyzed using BD FACSVerse™ Flow Cytometer (BD Biosciences, San Jose, USA). We used the species *Nicotiana tabacum* L. as the DNA reference standard and its genome size is 4,459 M (P1 in Supplementary Fig. S1a and Fig. S1b, Zonneveld *et al.*, 2005). The peaks of P2 in Supplementary Fig. S1a and Fig. S1b belong to *A. aureum* and *A. speciosum*, respectively. Based on the reference, the genome sizes of *A. aureum* and *A. speciosum* were estimated as 23.83 G and 23.75 G, respectively. Because the genome sizes of *A. aureum* and *A. speciosum* are almost the same and *A. aureum* has been reported as diploid with the chromosome number of 2n = 60 (Macron *et al*., 2003 and Lovis, 1997), we affirm that *A. speciosum* is a diploid species.

Transcriptome annotations

A BLASTX homolog search against the SwissProt database found matches to 19,304 *A. aureum* contigs, 17,002 *A. speciosum* contigs and 21,832 *C. thalictroides* contigs (Table 2). When a BLASTX search was performed against the NCBI non-redundant (NR) protein database, a total of 25,484 contigs in *A. aureum*, 21,250 in *A. speciosum* and 29,413 in *C. thalictroides* had hits (Table 2), of which 76.63%, 81.35% and 79.36% showed > 60% similarity, respectively (Supplementary Table S1 – S3, Supplementary Fig. S3). The top blast hit species were *Physcomitrella patens* and then *Selaginella moellendorffii* and *Picea sitchensis* (Supplementary Table S1 – S3, Supplementary Fig. S4). The retrieved GO terms based on the BLASTX against the NR for three ferns represented 12,100 (25.46%), 10,353 (28.43%) and 14,834 (24.39%) contigs, respectively (Table 2, Supplementary Table S4 – S6). The low annotation percentages may have resulted from the limited availability of fern transcriptomic and genomic resources in the public databases. A total of 4,501, 3,642 and 6,143 unigenes were assigned to 124, 122 and 127 KEGG (Kyoto Encyclopedia of Genes and Genomes) pathways for *A. aureum*, *A. speciosum* and *C. thalictroides*, respectively (Table 2, Supplementary Table S7), and GOanna in Agbase annotated 42.05% of *A. aureum* contigs, 45.63% of *A. speciosum* contigs and 38.95% of *C. thalictroides* contigs.

In the GO enrichment analysis, the most abundant GO terms of cellular component category were ‘cell part’

and ‘organelle’. The terms ‘catalytic activity’ and ‘binding’ were abundant in the molecular function category, whereas the most represented terms in the biological process category were ‘metabolic process’ and ‘cellular process’ (Fig. 1).

We downloaded the contigs of the six fern genomes (*Dipteris conjugata*, *Cystopteris protrusa*, *Plagiogyria formosana*, *Ceratopteris richardii*, *Pteridium aquilinum* and *Polypodium glycyrrhiza*) from Wolf *et al*. (2015) and queried three transcriptomes against them using BLASTN at an e-value cut-off of 10^{-3}. The results showed that 27,161 *A. aureum* contigs, 22,402 *A. speciosum* contigs and 48,256 *C. thalictroides* contigs had matches that accounted for 57.16% – 79.34% of all unigenes. A total of 17,471, 15,409 and 37,962 unigenes had blast hits longer than 100 bp (Supplementary Table S8).

Transcription factors (TFs)

We searched for transcription factors based on 1,770 Arabidopsis transcription factors downloaded from The *Arabidopsis* Gene Regulatory Information Server (AGRIS) (http://arabidopsis.med.ohio-state.edu/) using BLASTX with an e-value cut-off of 10^{-6}. We chose the best hit with an identity of more than 80%.

A total of 134, 126 and 136 TFs belonging to 24, 29 and 29 TF families, respectively, were identified in *A. aureum*, *A. speciosum* and *C. thalictroides*, respectively. AP2/EREBP, C2H2 and C2H2-Dof families were the most abundant in all three ferns (Supplementary Table S9). *A. speciosum* include more expressed members in the AP2/EREBP family than did the other two ferns.

Simple sequence repeats (SSRs)

Simple sequence repeats (SSRs or microsatellites) are a useful resource in the design of molecular primers for analyses of genetic diversity and gene mapping. MISA (MIcroSAtellite identification tool, http://pgrc.ipk-gatersleben.de/misa/) is a popular tool for SSR identification, and we used it to search the SSRs in the three fern transcriptomes. We set the search criteria at a minimum of five repeats of two to six nucleotides.

For *A. aureum* and *A. speciosum*, we identified 6,659 and 6,665 sequences containing 8,533 and 8,733 SSRs, respectively. However, only 4,536 SSRs in 3,784 sequences were identified for *C. thalictroides* (Supplementary Table S10). The mean SSR densities of *A. aureum*, *A. speciosum* and *C. thalictroides* were one per 4,076 bp, 4,177 bp and 7,740 bp, respectively. For the three transcriptomes, the most abundant repeat motif was di-nucleotide (82.68%, 82.13% and 86.42% for *A. aureum*, *A. speciosum* and *C. thalictroides*, respectively), followed by tri-nucleotide (16.42%, 17.03% and 12.21% for *A. aureum*, *A. speciosum* and *C. thalictroides*, respectively, Supplementary Table S10). The proportions of tetra-nucleotide, penta-nucleotide and hexa-nucleotide motifs were low in all three transcriptomes. The most dominant di-nucleotide repeat motif was AG/CT (63.77%, 65.87%, and 66.05%, respectively), and the lowest proportion di-nucleotide motif in the two *Acrostichum* species was AT/AT (2.23% and 2.73%) and in *C. thalictroides* was CG/CG (1.81%). Among the tri-nucleotide motifs, most in *C. thalictroides* were ATC/ATG (38.45%), whereas the most abundant repeat motif in the two *Acrostichum* species

was AGG/CCT (24.91% and 24.61% for *A. aureum* and *A. speciosum*, respectively) followed by AGC/CTG (22.20% and 20.98%, respectively, Supplementary Fig. S5). In summary, the three ferns showed a similar pattern in term of di-nucleotide motifs, whereas the two *Acrostichum* ferns differed from *C. thalictroides* in the proportions of tri-nucleotide motifs.

References:

Lovis, J. D. Evolutionary patterns and processes in ferns. *Adv. Bot. Res.* **4**, 229-415 (1978).

Marcon, A. B., Barros, I. C. L. & Guerra, M. A karyotype comparison between two closely related species of Acrostichum. *Am. Fern J.* **93**, 116-125 (2003).

Tindale, M. D. & Roy, S. K. A cytotaxonomic survey of the Pteridophyta of Australia. *Aust. Syst. Bot.* **15**, 839-937 (2002).

Zhang, R. *et al.* Molecular evidence for natural hybridization in the mangrove fern genus Acrostichum. *BMC Plant Biol.* **13**, 74 (2013).

Dolezel, J., Greilhuber, J. & Suda, J. Estimation of nuclear DNA content in plants using flow cytometry. *Nat. Protocols* **2**, 2233-2244 (2007).

Zonneveld, B., Leitch, I. & Bennett, M. First nuclear DNA amounts in more than 300 angiosperms. *Ann. Bot. (Lond).* **96**, 229–244 (2005).

Wolf, P. et al. An Exploration into Fern Genome Space. *Genome Biol Evol* **7** (2015).

Supplementary Figures

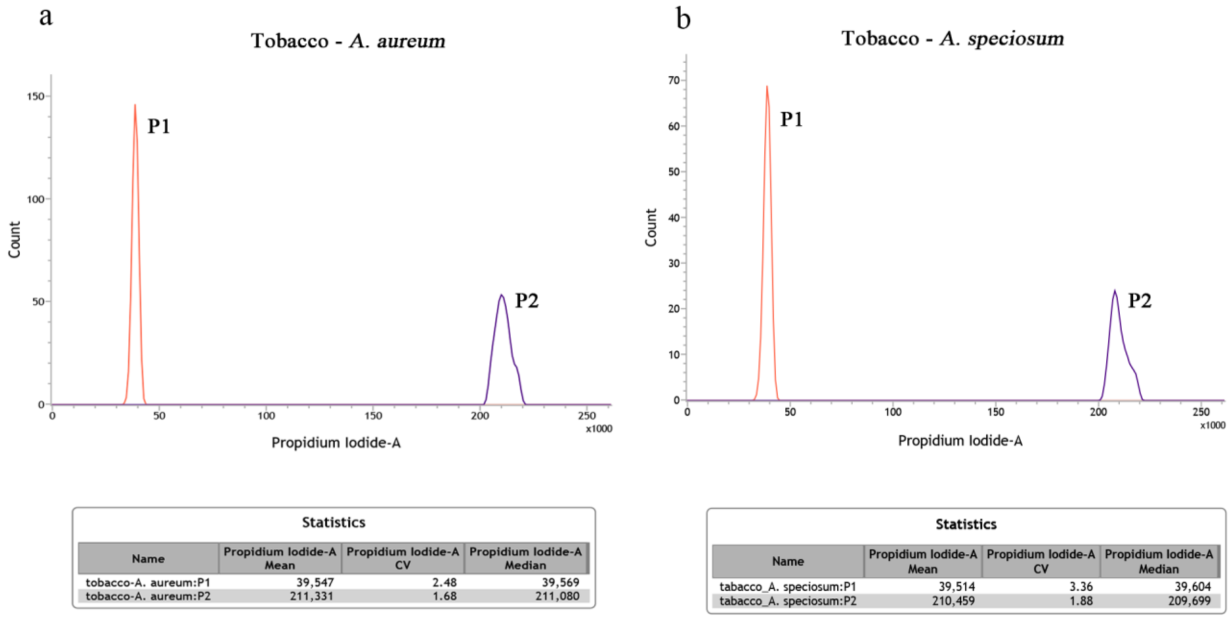

Statistics (a)

Name	Propidium Iodide-A Mean	Propidium Iodide-A CV	Propidium Iodide-A Median
tobacco-A. aureum:P1	39,547	2.48	39,569
tobacco-A. aureum:P2	211,331	1.68	211,080

Statistics (b)

Name	Propidium Iodide-A Mean	Propidium Iodide-A CV	Propidium Iodide-A Median
tabacco_A. speciosum:P1	39,514	3.36	39,604
tabacco_A. speciosum:P2	210,459	1.88	209,699

Supplementary Figure S1. The results of genome sizes for *A. aureum* (a) and *A. speciosum* (b). The P1 belongs to *Nicotiana tabacum* L., whereas the P2 in two panels refer to *A. aureum* and *A. speciosum*, respectively.

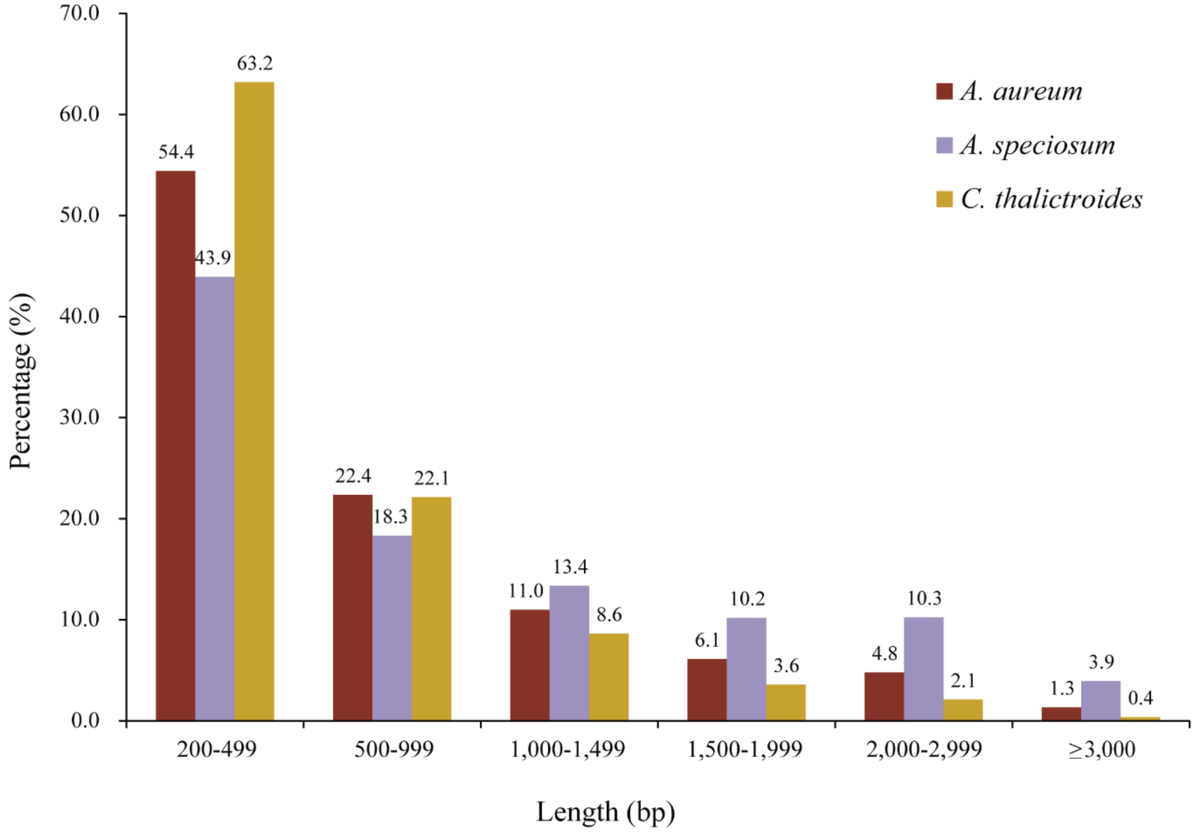

Supplementary Figure S2. Length distributions of *A. aureum, A. speciosum* and *C. thalictroides*. The percentage is shown above each bar.

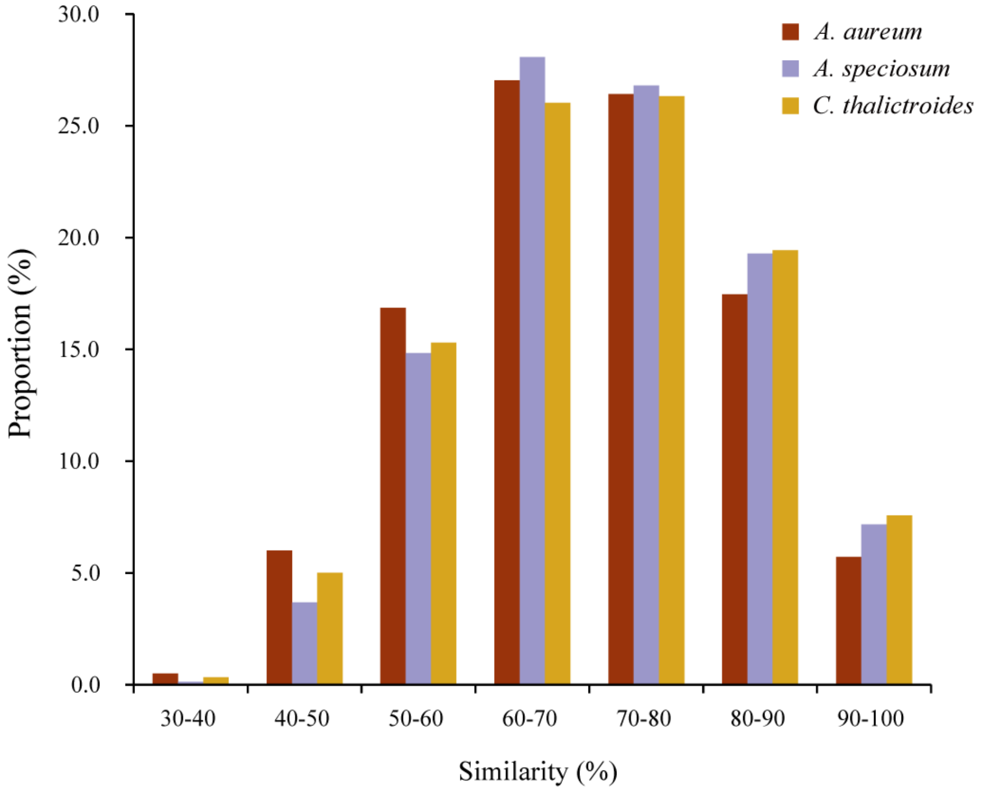

Supplementary Figure S3. Similarity distribution of blast hits against the NCBI NR database for unigenes of *A. aureum, A. speciosum and C. thalictroides*.

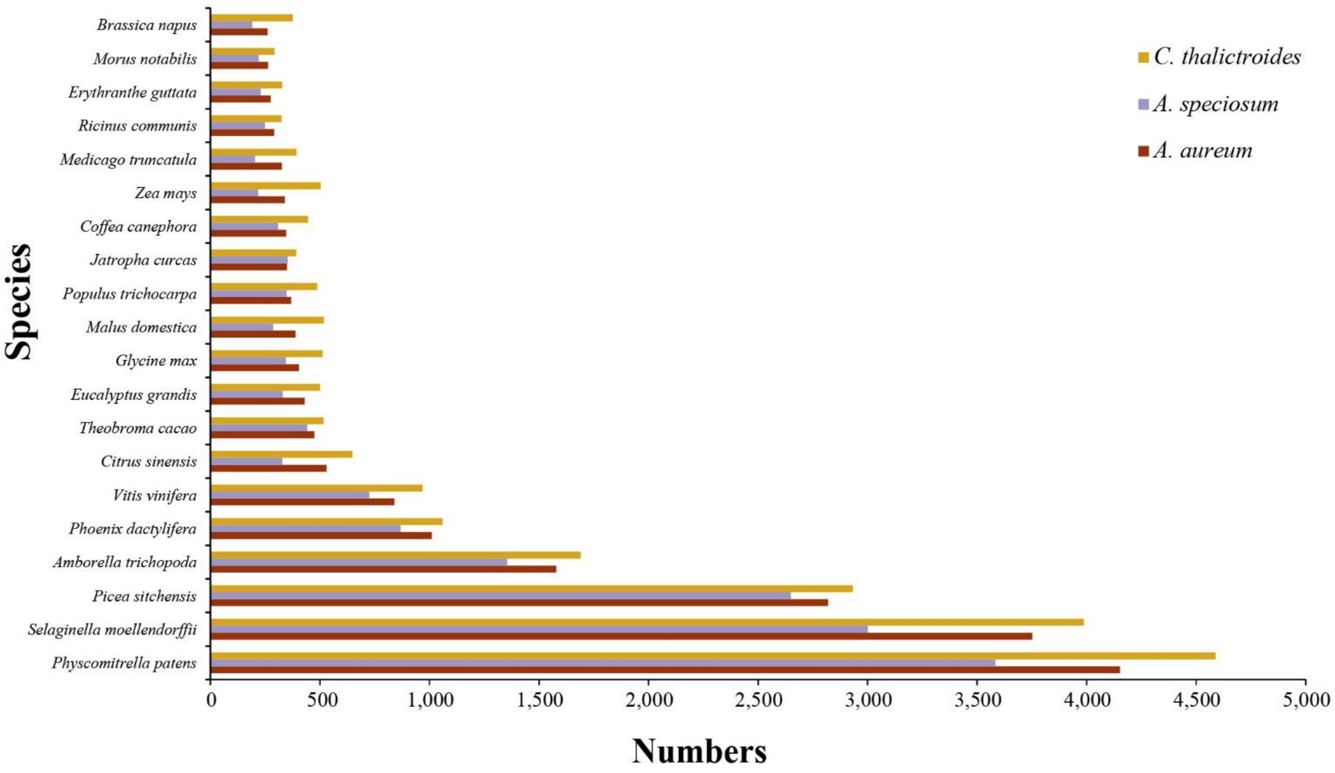

Supplementary Figure S4. Top-hit species against the NCBI NR database for unigenes of *A. aureum, A. speciosum* and *C. thalictroides*. The top 20 species are shown.

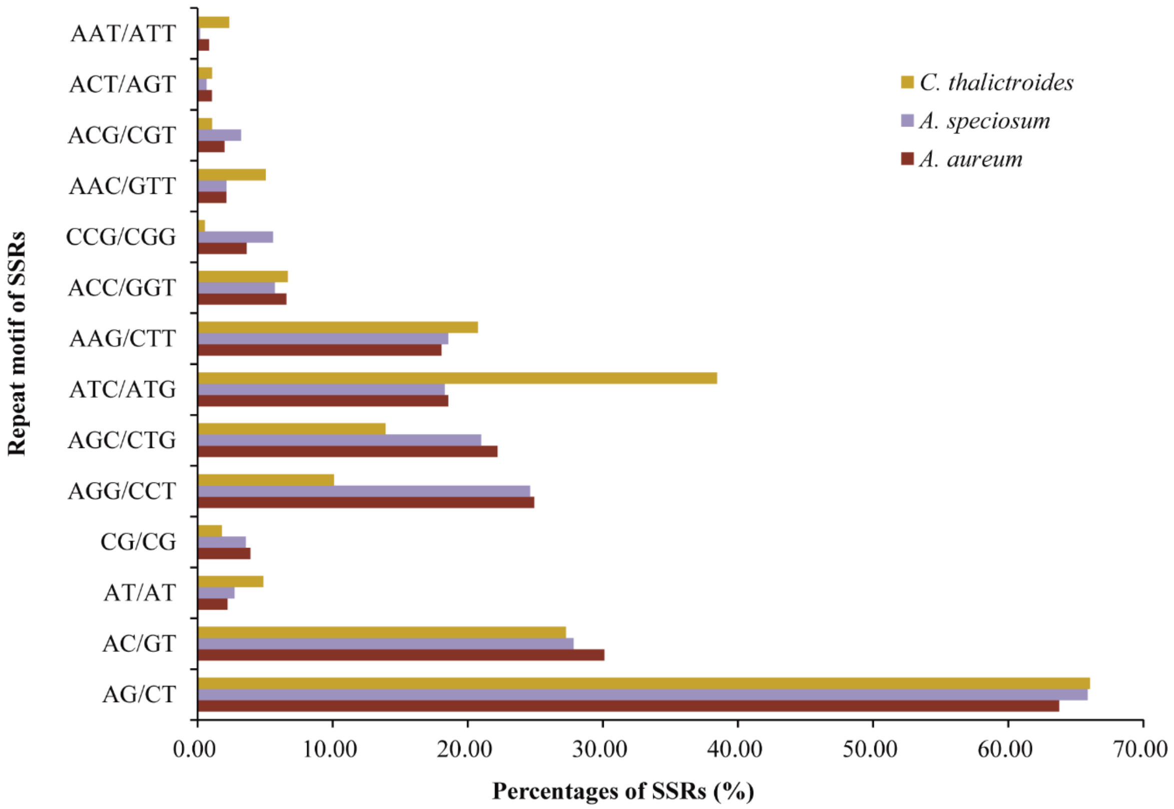

Supplementary Figure S5. Comparison of the percentages of different SSR repeat motifs for *A. aureum*, *A. speciosum* and *C. thalictroides*.

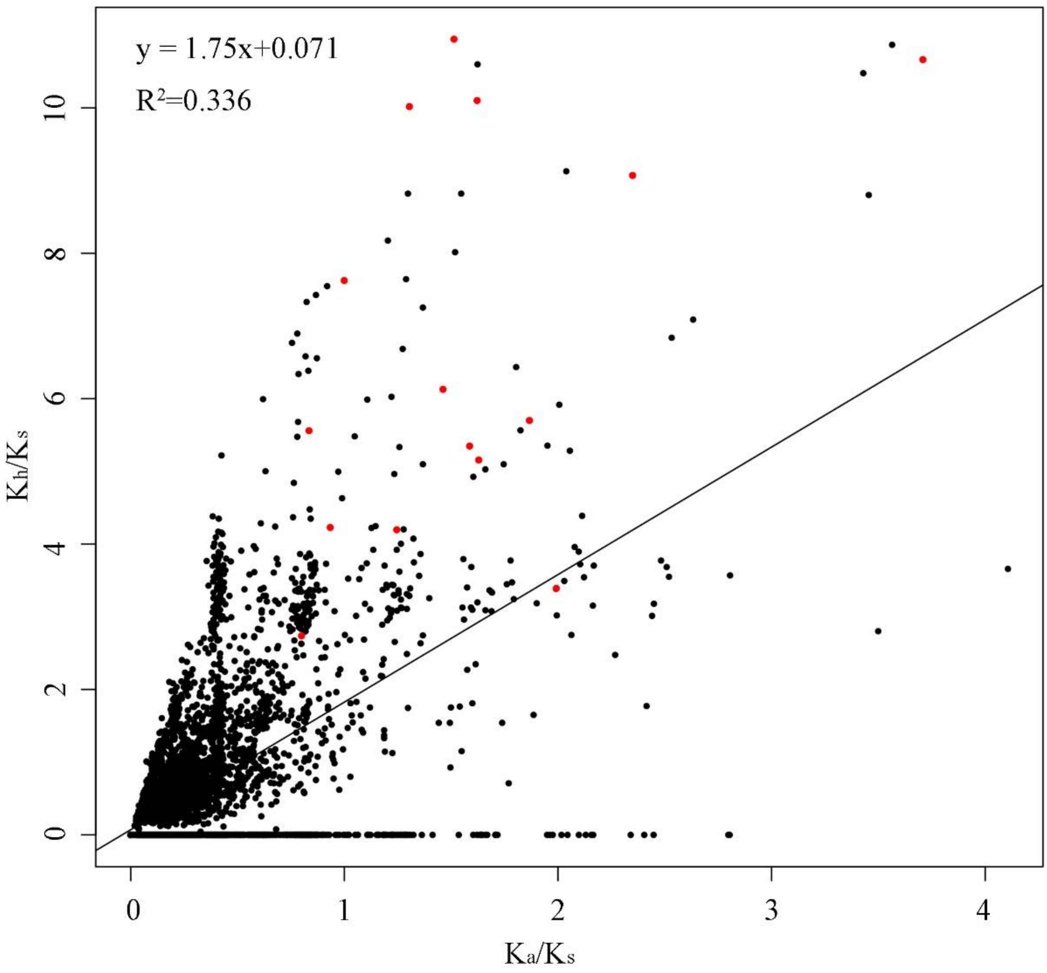

Supplementary Figure S6. Scatter plot of K_h/K_s versus K_a/K_s for each ortholog. The red dots indicate genes with K_h/K_s significantly greater than 1 (Fisher's exact test, $p < 0.05$).
